# Increased Monocyte Production of IL-6 after Toll-like Receptor Activation in Children with Autism Spectrum Disorder (ASD) Is Associated with Repetitive and Restricted Behaviors

**DOI:** 10.3390/brainsci12020220

**Published:** 2022-02-05

**Authors:** Heather K. Hughes, Charity E. Onore, Milo Careaga, Sally J. Rogers, Paul Ashwood

**Affiliations:** 1 M.I.N.D. Institute, University of California Davis, Sacramento, CA 95817, USA; hkahughes@ucdavis.edu (H.K.H.); ceonore@ucdavis.edu (C.E.O.); mcareaga@ucdavis.edu (M.C.); sjrogers@ucdavis.edu (S.J.R.); 2 Department of Medical Microbiology and Immunology, School of Medicine, University of California, Davis, CA 95616, USA; 3 Department of Psychiatry and Behavioral Sciences, University of California, Davis, CA 95616, USA

**Keywords:** autism, ASD, immune, inflammation, monocyte, toll-like receptors, IL-6, restricted

## Abstract

The prevalence of autism spectrum disorder (ASD) has starkly increased, instigating research into risk factors for ASD. This research has identified immune risk factors for ASD, along with evidence of immune dysfunction and excess inflammation frequently experienced by autistic individuals. Increased innate inflammatory cytokines, including interleukin (IL)-6, are seen repeatedly in ASD; however, the origin of excess IL-6 in ASD has not been identified. Here we explore specific responses of circulating monocytes from autistic children. We isolated CD14^+^ monocytes from whole blood and stimulated them for 24 h under three conditions: media alone, lipoteichoic acid to activate TLR2, and lipopolysaccharide to activate TLR4. We then measured secreted cytokine concentrations in cellular supernatant using a human multiplex bead immunoassay. We found that after TLR4 activation, CD14^+^ monocytes from autistic children produce increased IL-6 compared to monocytes from children with typical development. IL-6 concentration also correlated with worsening restrictive and repetitive behaviors. These findings suggest dysfunctional activation of myeloid cells, and may indicate that other cells of this lineage, including macrophages, and microglia in the brain, might have a similar dysfunction. Further research on myeloid cells in ASD is warranted.

## 1. Introduction

Autism spectrum disorder (ASD) is a heterogenous disorder characterized by impairments in social interactions and communication, accompanied by restricted and repetitive behaviors of varying degrees [1]. An alarming increase in the prevalence of ASD has occurred over the past several decades. Recent estimation of this prevalence is currently 1 in 59 children, with males having a significantly higher prevalence than females [1]. Research efforts have sought to determine if this rise is purely due to better recognition of the condition or whether individual or combined environmental and/or genetic risk factors are responsible for this increase. Substantial comorbidities are seen in ASD, including gastrointestinal dysfunction, seizures, and sleep disorders [2]. These contribute to decreasing quality of life for autistic individuals; therefore, identifying and treating biological factors driving these complex disorders could help alleviate some of these morbidities and improve outcomes for autistic individuals and their families. One biological factor that has been hypothesized as contributing to the etiology and pathophysiology of ASD is immune system abnormalities that may be driving deviant neuroimmune responses [3].

An abundance of evidence supports the hypothesis of the immune system contributing to ASD (reviewed in [4]). This evidence includes genetic associations, including an increased familial risk of autoimmunity in ASD, and several candidate genes involved in immune function and regulation that been identified in ASD. For example, human leukocyte antigen (HLA) locus variants are associated with ASD and other neurodevelopmental disorders, including alleles that encode the major histocompatibility complexes I and II and complement C4b-null [5]. Other candidate genes with immune involvement include *MET*, macrophage migration inhibitory factor (*MIF*), and mutations in the interleukin (IL)-1 receptor family [6,7,8,9], among others. Inflammatory conditions during gestation also substantially increase the risk of having a child with ASD, and infection during pregnancy leading to maternal immune activation (MIA) has been identified as a significant risk factor [10,11,12,13]. This has been supported by animal models of MIA, which have identified mechanisms that include exposure to inflammatory cytokines including IL-6 and IL-17 during gestation that may be leading to fetal immune priming, and provide support for aberrant innate immune function leading to ASD-relevant behaviors [14,15,16,17,18,19,20]. Significant immune dysfunction in individuals with ASD is also a prominent finding [4]. Several studies have identified increases in circulating inflammatory cytokines associated with the innate immune system in ASD [21,22,23,24], along with a reduction in aspects of immune regulation [25,26,27] suggesting dysfunction of the innate immune system, which initiates downstream immune responses. However, these findings are sometime inconsistent as some studies have seen decreased plasma cytokines/chemokines in ASD [28].

When exposed to a pathogen or other immune insult, the first line of defense—the innate immune system—is activated. Cellular components of the innate arm include neutrophils, monocytes and their tissue-resident counterparts, the macrophages. These cells express pattern-recognition receptors (PRRs) including Toll-like receptors (TLRs) that broadly recognize pathogen-associated molecular patterns (PAMPs). This recognition triggers a cascade of activation that leads to inflammatory responses and the release of canonical innate inflammatory cytokines, namely IL-1β, IL-6 and a tumor necrosis factor (TNF)α, during the initial and early immune response [29]. Innate myeloid cells such as monocytes and macrophages contribute substantially to the production of these inflammatory cytokines. Although many studies have identified increases in inflammatory cytokines in ASD, few have isolated and activated circulating monocytes to identify dynamic responses of myeloid cells in ASD children, and of those, results have been mixed. We previously found increased expression of CD95, a receptor upregulated on activated cells, in resting CD14^+^ monocytes from ASD children [30]. Further exploration found inflammatory activation of CD14^+^ monocytes in young children with ASD after TLR stimulation [31]. More recently, we found that activation of TLRs on isolated monocytes from ASD children at a slightly later age led to dysregulated gene expression associated with inflammation and regulation of translation [32]. Innate cellular immune responses at this later age have not been explored.

Our research sought to identify if aberrant inflammatory immune responses and increases in inflammatory cytokine production seen after TLR activation of CD14^+^ monocytes in young ASD children would be recapitulated, and if dysregulated responses correlated with behavior. We found that after TLR4 stimulation, IL-6 production was significantly increased in ASD monocytes, and this increase was associated with repetitive behaviors. Our results suggest that immune dysfunction in ASD may shift only slightly over time and still produce a robust innate inflammatory profile. Our findings strengthen the link between inflammatory cytokines and behavioral severity in ASD.

## 2. Materials and Methods

### 2.1. Study Participants

Enrollment for this research took place as part of the Autism Phenome Project (APP) study at the UC Davis M.I.N.D. Institute. Detailed recruitment protocols for APP have been described previously [33,34]. Experienced specialists at the UC Davis M.I.N.D. Institute administered the diagnostic instruments used in this study. Briefly, participants were clinically evaluated for placement into one of two diagnostic groups, with final diagnosis confirmed by the Autism Diagnostic Interview-Revised (ADI-R) [35] and the Autism Diagnostic Observation Schedule (ADOS) [36]: (1) children diagnosed with ASD [*n* = 25, 19M/6F, median age 5.62 (interquartile range 5.08–6.35) years]; or (2) children with typical development (TD) [*n* = 20, 16M/4F, 5.48 (4.86–6.13) years]. Inclusion criteria for TD controls included scoring that fell within two standard deviations of mean on the Mullen’s Scale of Early Learning (MSEL). TD children were also screened for communication deficits and excluded for ASD (scores > 15) on the Lifetime version of the Social Communication Questionnaire [37]. Additional developmental and behavioral testing included the Repetitive Behaviors Scale—Revised (RBS-R) [38]. The RBS-R was used to assess a number of subdomains including: (a) Stereotypic Behavior; (b) Self-injurious Behavior; (c) Ritualistic Behavior; (d) Sameness Behavior; and (e) Restricted Interests. See Supplemental Table S1 for summarized demographic information and clinical scores.

TD children were excluded from the study if diagnosed with a language impairment, or any known behavioral, neurological, or developmental problems. Regardless of group, participants were excluded if diagnosed with psychiatric disorder(s) or other neurological disorders (i.e., Fragile X, etc.), and/or seizure disorder. Participants with immune mediated disorders including autoimmune disease, celiac disease, or inflammatory bowel disease were also excluded. Parent interviews took place to screen for additional exclusion criteria including prescription medication use, recent illness or fever (within the last two days). Participants were ambulatory, had no hearing or vision problems, and were native English speaking. All participants’ parents/guardians gave informed consent. This study was approved by the institutional review boards at the University of California, Davis.

### 2.2. Cell Isolation

Blood was drawn and collected into an acid-citrate-dextrose Vacutainer tube (BD Biosciences; San Jose, CA, USA) then centrifuged for 10 min at 2300 rpm within 12 h of collection. Plasma was removed and stored at −80 °C. The remaining pellet containing red and white blood cells was brought up to 35 mL in Hanks Balanced Salt Solution (HBSS; Gibco, Gaithersburg, MD, USA) without Ca^2+^ or Mg^2+^, then carefully layered over a Ficoll-Paque gradient (Pharmacia Biotech, Piscataway, NJ, USA). Layered blood was centrifuged at room temperature for 30 min. at 1700 rpm. The interface layer containing peripheral blood mononuclear cells (PBMC) was collected and washed twice with HBSS. CD14^+^ cells were isolated using an anti-CD14 magnetic bead according to the manufacturer’s protocol (Miltenyi). Positive selection of CD14^+^ was chosen based on our previous work that identified >95% purity and less activation using this method, compared to >85% purity and greater activation (likely due to dendritic cell contamination) with negative selection [39]. Trypan blue exclusion was used to determine concentration of live cells. Cells were brought up to their working concentration in RPMI 1640 (Gibco) with 10% low endotoxin heat-inactivated fetal bovine serum (Omega Scientific; Tarzana, CA, USA), 100 IU/mL penicillin, and 100 IU/mL streptomycin (Sigma, St Louis, MO, USA) added.

### 2.3. Cellular Stimulations

An amount of 1 × 10^5^ CD14^+^ cells in RPMI were seeded per well into 96-well cell culture plates and incubated for 24 h at 37 °C in either RPMI media alone, RPMI with 1 µg/mL lipopolysaccharide (LPS; *Escherichia coli* serotype 0111:B4, Sigma, St. Louis, MO, USA), or 10 µg/mL lipoteichoic acid (LTA; *Staphylococcus aureus*, Sigma, St. Louis, MO, USA). Culture conditions included 5% CO_2_. The timing of stimulation was based on prior studies indicating that 24 h stimulation was optimal for monocyte production of innate inflammatory cytokines. After incubation period was over, the plates were centrifuged at 2000 rpm for 10 min after which time supernatant was collected from each well and stored at −80 °C.

### 2.4. Luminex Multiplex Analysis

Supernatants from CD14^+^ cellular cultures were quantified by multiplex Luminex™ 100 suspension array system (Bio-Plex 200; Bio-Rad Laboratories, Hercules, CA, USA) per the manufacturer’s instructions, to detect the presence and concentrations of cytokines and chemokines commonly produced by monocytes. These methods have been described in detail in previous studies [40]. Briefly, antibody-coupled beads were added to 25 µL of supernatant and incubated then washed to remove unbound beads. They were next incubated with biotinylated detection antibody followed by the addition of streptavidin–phycoerythrin. Sample concentrations were analyzed by Luminex and concentrations were determined by Bio-Plex Manager software using a standard curve derived from serial dilutions of reference cytokine concentrations included in the assay, with final concentrations calculated using a five-parameter model. Assay limits of detection (LOD) were: GM-CSF (2.3 pg/mL), IL-1β (0.7 pg/mL), IL-6 (0.4 pg/mL), IL-10 (0.3 pg/mL), IL-12(p40) (12.3 pg/mL), IL-12(p70) (0.9 pg/mL), IP-10 (1.3 pg/mL), MCP-1 (1.2 pg/mL), and TNFα (0.2 pg/mL). One-half of the LOD was used to replace values below the limit of detection for statistical analysis.

### 2.5. Statistical Analysis

Shapiro–Wilks test determined that the majority of cytokine data were not normal; therefore, groups were compared using the non-parametric Mann–Whitney U test. Corrections for multiple comparisons were achieved by Holm–Sidak method. Correlation analysis was performed with Spearman analysis. A *p* value of less than 0.05 was considered statistically significant. Data analyses were performed with SPSS Statistics Version 27 (IBM, Armonk, NY, USA) and GraphPad Prism Version 9.0 (GraphPad Software, San Diego, CA, USA).

## 3. Results

Secreted cytokines were measured in supernatant collected from unstimulated and stimulated (LPS and LTA) monocytes after 24 h of culture. In media alone, no differences were seen in cytokine production from ASD or TD monocytes (Table 1). The Mann–Whitney U test revealed that ASD monocytes produced significantly more IL-6 when activated with the TLR-ligand LPS (median concentration: 18,059 pg/mL, *n* = 24) compared to LPS-stimulated monocytes from TD children (12,881 pg/mL, *n* = 19; U = 116.5, *p* ≤ 0.0056, Table 1). ASD monocyte IP-10 concentrations were also increased after LTA stimulation (6.69 pg/mL, *n* = 20) compared to TD monocytes (2.61 pg/mL, *n* = 16, U = 96.5, *p* = 0.0428); however, this increase did not remain significant after multiple testing correction (adjusted *p* = 0.3254). After Spearman’s correlation analysis, IL-6 production after LPS stimulation was associated with increased repetitive behaviors (Table 2). No differences in responses between males and females were seen (data not shown); however, the sample size of female participants was small.

## 4. Discussion

Few studies have investigated the cellular responses from stimulated monocytes isolated from autistic children. We previously identified dysregulated CD14^+^ monocyte responses after TLR activation in very young children with ASD [31], while other studies identified differences in monocyte responses after the stratification of groups by clinical measures of asthma/allergy [41,42]. In our current study, we extended our previous findings to a later timepoint and found that LPS-activated monocytes produce significantly higher IL-6 than monocytes from ASD children compared to typically developing children. This suggests that monocyte activation is dysfunctional in ASD, persists over time, and may indicate an inability to appropriately regulate an immune response after activation. This may be leading to increased and sustained concentrations of inflammatory cytokines, namely IL-6, a cytokine implicated in ASD. Moreover, we saw significant correlations of IL-6 to worsening behaviors, specifically those involving repetitive behavior.

IL-6 is a pleiotropic inflammatory cytokine involved in early innate immune responses. It is one of the main inducers of inflammatory responses, driving the production of acute phase proteins and leading to an inflammatory activation cascade that drives downstream adaptive immune responses, including T cell activation and expansion [43]. Appropriate amounts of cytokines, including those inflammatory in nature, are involved in brain and central nervous system (CNS) development under tightly regulated conditions (reviewed in [44]). IL-6 stimulates differentiation and growth of astrocytes, neurons, and Schwann cells [45,46,47]; however, culture conditions of excess IL-6 lead to neuronal loss, suggesting that concentrations must be ideal for the proper development and homeostasis of neurons [48]. Human studies have provided recent insight that variations in exposure to IL-6 during gestation alters structural and functional connectivity in the brain, and influences executive function, working memory and cognition [49,50,51]. Maternal inflammation during gestation confers an increased risk of ASD in the child and IL-6 has been implicated in ASD animal models of maternal infection and immune activation [11,52,53].

Innate immune cells such as monocytes are major producers of IL-6. Although not identical, monocytes share similarities with tissue-resident macrophages including microglia, the innate cells of the CNS and brain. In addition to being first-line defenders in the CNS, these myeloid-derived cells are critical for homeostasis in the brain. They have inflammatory capabilities when activated, and produce IL-6 under inflammatory conditions [54]. Genes associated with microglial activation are upregulated in the postmortem ASD brain [55,56,57]. This activation, along with recruitment of circulating monocytes/macrophages, could be responsible for increased IL-6 and aberrant findings seen in the postmortem ASD brain. For example, loss of neurons along with evidence of infiltrating macrophages and increased inflammatory cytokines has been seen in the postmortem ASD brain. In this study, protein array results found a 31.4-fold increase in IL-6 in the anterior cingulate gyrus along with increased MCP-1, a chemokine responsible for macrophage recruitment. Elevations of inflammatory cytokines were also seen in the cerebrospinal fluid (CSF) [58]. Li, et al. found increased TNFα, IL-6, IL-8, and GM-CSF in the postmortem ASD brain compared to tissue from age-matched controls [59]. This group later found IL-6 significantly increased in the cerebellum of post-mortem ASD brain. When exploring possible mechanisms of impairment by elevations of IL-6 in the ASD brain, they found that IL-6 impaired adhesion and cell migration. IL-6 also promoted the increased formation of excitatory synapses in cerebellar granule cells, but no changes in inhibitory synapse formation [60]. The improper formation of synapses, and imbalances in excitation and inhibition, are implicated in neurodevelopmental disorders, including ASD [61,62]. Gene expression profiling of ASD temporal cortex identified increased expression in genes associated with several inflammatory immune pathways, namely IL6, NFKB, IL1B, IMFLAM, and TOLL [63]. Together, these studies suggest aberrant immune responses in the CNS may be contributing mechanistically to ASD, with myeloid-derived cells including microglia and infiltrating monocytes/macrophages playing a role.

Increased IL-6 is a repeated finding in ASD. For example, autistic children exhibited increased plasma IL-6, along with elevated IL-1β, IL-8, and IL-12p40. Increased inflammatory cytokines were also associated with worse behavioral scores, and increased stereotypy, significantly associated with IL-6 concentrations [21]. Similar findings were then seen in an all-male cohort of ASD individuals, with plasma IL-1β, IL-12(p70), IL-8, and GRO-a (CXCL1) increased along with T helper cell associated cytokines, IL-5, IL-13, and IL-17 [22]. Ricci et al. found increased serum IL-1β, IL-6, IL-23, IL-12 and TNFα in ASD over a wide age range [23]. Elevated serum ENA-78 (CXCL5), an innate chemokine produced under inflammatory conditions by stimulation with IL-1β and TNF-α, has also been identified in ASD children [24]. Reductions in the regulatory cytokine TGFβ1 may be contributing to an aberrant innate immune response. This has been seen in plasma, sera, and after cellular activation in autistic individuals, with associations to worsening behaviors [25,26,27,64]. Aberrant cellular responses associated with innate immunity and leading to increased IL-6 has also been seen in ASD. An early study found increased PBMC production of IL-6 after activation of TLR4 in some ASD individuals; however, there was high variability among subjects [65]. Increased IFN-γ and IL-1RA seen in whole blood cultures suggests the activation of peripheral monocytes [66]. We previously found increased IL-6 among other inflammatory cytokines after TLR activation on monocytes in very young children with ASD [31]. This current study repeated this experiment at a slightly later timepoint, and identified IL-6 increases after TLR4 activation. Our findings of increased IL-6 after monocyte activation in ASD also suggest that circulating monocytes are, at least in part, responsible for excess innate inflammatory cytokines seen in ASD plasma/serum.

We previously explored gene expression of these cells after LPS and LTA stimulation and found an increased expression of inflammatory genes in ASD monocytes, including increased NFκB1 which could be driving increased IL-6 production. We also found that genes associated with translation were dysregulated in ASD monocytes, with a dampening in expression of TD monocyte translation-associated genes not seen in ASD monocytes [32]. This dysfunctional regulation could provide an alternate explanation as to the increased IL-6 seen at the protein level, as lack of appropriate regulation of translation could lead to excess cytokine protein production. In this study IP-10 was also elevated; however, this was only seen after LTA stimulation and this increase was not significant after correcting for multiple comparisons. Elevated IP-10 has been seen previously in ASD in the CSF which potentially reflects activity of microglia [58]. However, peripheral levels of IP-10 are generally decreased or unchanged in ASD [22,67,68,69]. Peripheral cytokines are influenced by a number of cell types, not just monocytes. Additionally, peripheral plasma/serum studies are a snapshot of the immune status of the individual at time of blood draw, and do not necessarily reflect how various immune cells will behave under conditions of activation. When studies do involve activated immune cells, they are usually PBMC which include cells of both the innate and adaptive arms of the immune system that may be influencing the behavior of each other under culture conditions. The differing dynamics of autocrine and paracrine signaling of the monoculture compared to a multicellular culture may explain why we only saw IL-6 and IP-10 elevations in these cells, contrary to other cytokine studies in ASD. Our study focused on whether monocyte-specific responses to TLR activation differ in ASD, helping us pinpoint very specific TLR signaling induced responses of monocytes in ASD. The increased IL-6 and IP-10 seen in this study are unlikely to be attributed to increased TLR expression on ASD monocytes. We previously found no differences in TLR2 or TLR4 protein levels on monocytes by flow cytometry (TLR4 data not shown) [31]. Additionally, when examining gene expression in ASD monocytes, no genes encoding TLRs were differentially expressed compared to TD monocytes, either before or after stimulation with LPS or LTA [32]. Furthermore, we are unaware of any study that has identified increases in TLR2/4 expression in ASD monocytes, and increases in these receptors would have led to a more global cytokine response, including increases in IL-1β, TNFα, IL-12, and others. However, additional investigations of TLR receptor expression and signaling molecules downstream of TLR activation would be warranted in future studies.

Our study has several limitations, including small sample size and limited recruitment of female participants. The 24 h timepoint aligned with our previous study for the purposes of replication; however, we were limited by the number of cells isolated, preventing analysis of multiple timepoints after stimulation. At 24 h, we may be missing earlier and/or later responses, including those that regulate the immune response after inflammatory activation. Future investigation of multiple timepoints can help elucidate when during the immune response ASD monocytes are most dysregulated. Regardless of our limitations, this study adds strength to the growing evidence that inflammatory cytokines contribute to aberrant behaviors and may point to a cellular origin of excess IL-6 in ASD.

## Figures and Tables

**Table 1:** Monocyte cytokine production under each of three conditions. Table includes concentrations of cytokines produced at baseline (unstimulated), or after LPS or LTA stimulation, with data presented as median (IQR) pg/mL. Cytokine concentrations produced by TD monocytes were compared to concentrations produced by ASD monocytes under each condition. After correcting for multiple comparisons, monocytes from autistic children had a significant increase in IL-6 production after LPS stimulation.

**Unstimulated**	**TD (*n* = 20)**		**ASD (*n* = 25)**		***p* Value**	**Adjusted**	**U Value**
	**Median**	**IQR**	**Median**	**IQR**		***p* Value**	
GM-CSF	*b.d*.	*-*	*b.d.*	*-*	*-*	*-*	*-*
IL-1β	3.81	(2.44–9.39)	3.78	(2.12–8.73)	0.7469	0.9153	235.5
IL-6	17.3	(7.70–54.5)	18.7	(7.92–70.5)	0.709	0.9153	233
IL-10	2.04	(1.30–3.89)	2.53	(1.88–5.71)	0.2089	0.7549	194.5
IL-12p40	*b.d*.	*-*	*b.d.*	*-*	*-*	*-*	*-*
IL-12p70	*b.d*.	*-*	*b.d.*	*-*	*-*	*-*	*-*
IP-10	10.9	(6.32–22.0)	14.2	(9.77–31.9)	0.2444	0.7549	198.5
MCP-1	133.3	(66.2–267.8)	176.6	(97.3–465.4)	0.4345	0.88	215
TNFα	9.21	(6.55–18.2)	12.2	(6.66–20.2)	0.4114	0.88	213.5
**LPS Stimulated**	**TD (*n* = 19)**		**ASD (*n* = 24)**		***p* Value**	**Adjusted**	**U Value**
	**Median**	**IQR**	**Median**	**IQR**		***p* Value**	
GM-CSF	686	(433.0–987.1)	521.2	(285.9–844.6)	0.0803	0.4881	156
IL-1β	18,922	(8303–28,341)	17,546	(9570–28,341)	0.9666	0.9994	235.5
IL-6	12,881	(10,600–14,473)	18,059	(12,678–29,422)	0.0056 *	0.0493 *	116.5
IL-10	9424	(7586–12,616)	10,790	(7384–14,943)	0.8182	0.9989	218
IL-12p40	76.1	(56.2–145.4)	63.6	(32.4–131.9)	0.6324	0.9933	208
IL-12p70	6.16	(1.90–7.68)	4.32	(2.46–7.35)	0.918	0.9994	223.5
IP-10	42.9	(14.5–72.9)	49.2	(23.8–130.4)	0.1227	0.6000	164.5
MCP-1	9795	(9017–11,316)	9997	(9329–11,982)	0.2287	0.7895	178
TNFα	7535	(4141–9537)	6367	(5072–9700)	0.9903	0.9994	227
**LTA Stimulated**	**TD (*n* = 16)**		**ASD (*n* = 20)**		***p* Value**	**Adjusted**	**U Value**
	**Median**	**IQR**	**Median**	**IQR**		***p* Value**	
GM-CSF	178.6	(74.1–286.6)	96	(62.6–186.3)	0.1402	0.7013	113
IL-1β	3581	(2880–6472)	4393	(3699–5195)	0.5819	0.9947	142
IL-6	12,492	(10,858–15,598)	14,524	(11,604–20,300)	0.2204	0.825	121
IL-10	5154	(4017–5804)	5263	(3939–7693)	0.8136	0.9947	152
IL-12p40	493.9	(358.4–670.0)	436.1	(285.7–937.3)	0.9999	0.9999	160
IL-12p70	3.59	(2.26–5.64)	3.68	(1.94–4.38)	0.6078	0.9947	143.5
IP-10	2.61	(1.77–11.4)	6.69	(4.15–14.9)	0.0428 *	0.3254 ^†^	96.5
MCP-1	10,028	(9088–11,150)	10,105	(9357–11,244)	0.5819	0.9947	142
TNFα	8574	6239–9957)	6857	(4470–10,071)	0.6037	0.9947	143

*b.d.* = below detection; * *p* < 0.05, ^†^ significant prior to multiple testing correction.

**Table 2:** Repetitive behavior scale correlations to LPS-induced IL-6 responses. Spearman’s rank correlation analysis identified significant correlations between IL-6 levels and Repetitive Behavior Scale scores. Increased RBS score indicates worsening behavior. *ρ* = Spearman’s rho.

Repetitive Behavior Scale Correlations to IL-6	*p* Value	*ρ*	*n*
Stereotyped Behavior: total subscale score	0.002	0.470	40
Self-injurious Behavior: total subscale score	0.013	0.392	40
Ritualistic Behavior: total subscale score	0.009	0.407	40
Sameness Behavior: total subscale score	0.041	0.325	40
Restricted Behavior: total subscale score	0.014	0.385	40
Repetitive Behavior Scale: overall score	0.000	0.516	40

## Data Availability

The data presented in this study are available on reasonable request from the corresponding author.

## Supplementary Materials

The following supporting information can be downloaded at: https://www.mdpi.com/article/10.3390/brainsci12020220/s1, Table S1: Clinical characteristics and demographics of study participants.
